# Dopamine Reduction in the Substantia Nigra of Parkinson's Disease Patients Confirmed by In Vivo Magnetic Resonance Spectroscopic Imaging

**DOI:** 10.1371/journal.pone.0084081

**Published:** 2014-01-08

**Authors:** Adriane Gröger, Rupert Kolb, Rita Schäfer, Uwe Klose

**Affiliations:** Department of Diagnostic and Interventional Neuroradiology, Magnetic Resonance Research Group, University Hospital Tübingen, Tübingen, Germany; St. Jude Children's Research Hospital, United States of America

## Abstract

**Objectives:**

Metabolic changes in the substantia nigra of patients with Parkinson's disease were previously investigated in different molecular-pathological examinations. The aim of our study was the in vivo measurement of these alterations using three-dimensional magnetic resonance spectroscopic imaging.

**Methods:**

21 patients with Parkinson's disease and 24 controls were examined using magnetic resonance spectroscopic imaging at 3 Tesla. The spectra of rostral and caudal substantia nigra regions were analyzed using LCModel. For spectral fitting, an adjusted basis data set with pathology-specific metabolites and macromolecules was used to better reproduce the in vivo spectra. To assess differences between both groups more accurately, especially in metabolites at lower concentrations, group-averaged spectra were evaluated in addition to the analysis of individual data.

**Results:**

We found significantly decreased N-acetylaspartate, choline, creatine, *myo*-inositol, glutathione and dopamine concentrations in patients with Parkinson's disease compared to controls, whereas glutamine+glutamate, γ-aminobutyric acid, and homovanillic acid were slightly increased. According to anatomical features, clear differences in the biochemical profiles were found between rostral and caudal substantia nigra voxels in both groups.

**Conclusions:**

Reduced N-acetylaspartate and dopamine concentrations result from progressive degeneration of dopamine-producing neurons within the substantia nigra pars compacta. Decreased creatine levels can be interpreted as impaired energy metabolism due to mitochondrial dysfunction. Lower glutathione concentrations might be a cause or consequence of oxidative stress. Furthermore, slightly increased glutamine+glutamate and γ-aminobutyric acid levels are expected based on *post mortem* data in Parkinson's disease. To the best of our knowledge, this is the first non-invasive confirmation of these metabolic changes.

## Introduction

Parkinson's disease (PD) is a frequently occurring, age-related, neurodegenerative disorder causing bradykinesia, tremor and motor impairment. It is caused by progressive loss of dopamine-secreting neurons within the substantia nigra (SN) pars compacta [1]. In a post mortem study [2], the neurochemical markers of inhibitory and excitatory neurotransmission were measured within the SN. However, the process of neuron loss is still not well understood. There is strong evidence that mitochondrial dysfunction and oxidative stress play a causal role in PD pathogenesis, and discovering metabolic alterations in the SN would improve early diagnosis and could lead to therapeutic advances.

Magnetic resonance spectroscopic imaging (MRSI) is a widely used non-invasive method that provides information about metabolic composition, especially in MR spectra acquired at high magnetic field strengths and with short echo times, in which various metabolites can be detected. To estimate metabolite concentrations, the LCModel algorithm is most commonly used [3], [4]. However, methodological limitations such as complex multiplet resonance patterns, overlapping metabolites, variations in line shape, and underlying baseline variations make it difficult to quantify metabolites with lower concentrations and to confirm post-mortem results in individual spectra. In particular, it has not yet been possible to identify the dopamine signal in in-vivo spectra of Parkinson patients.

The goal of our study was the non-invasive measurement of metabolic changes within the SN of PD patients using 3D MRSI to confirm molecular-pathological post-mortem results in vivo. For this we used an improved LC-Model analysis and evaluated averaged spectra from groups of patients and controls.

## Materials and Methods

### Ethics Statement

All subjects gave their written informed consent to the MRSI examination before participating in this study, which was approved by the local ethics committee (Ethik-Kommission an der Medizinischen Fakultät der Eberhard-Karls-Universität und am Universitätsklinikum Tübingen) and adhered to institutional guidelines.

### Subjects

Examined subjects were characterized in a previous study by Gröger et al. [5]. In detail: 21 PD patients, aged between 54 and 82 years, with disease durations between 3 and 13 years and 24 neurologically healthy controls in the same age range (52–79 years) were investigated using 3D MRSI. Initial high-resolution MRI of all subjects were obtained and examined by a neuroradiologist to exclude brain morphological abnormalities.

### Data acquisition

**Figure 1:**
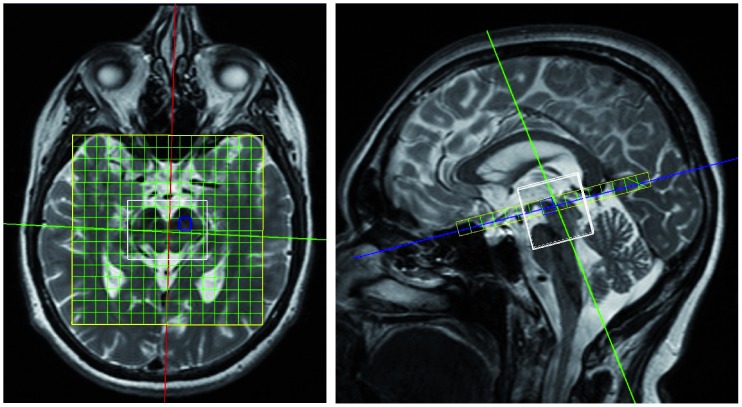
3D MRSI voxel localization in SN region for rostral slice.

3D MRSI was performed on a 3T MR scanner (Magnetom Tim Trio, Siemens Healthcare, Erlangen) with a 32-channel head coil. The protocol was previously specified by Gröger et al. [6]. In brief, 3D MRSI was achieved using a PRESS sequence (TE/TR = 30/1350 ms) with water saturation. The volume of interest was fitted to the size of the midbrain so that the SN region was located in the same voxels for all subjects. The resulting nominal voxel size was 6×6×7 mm^3^ so that two enclosed voxels (rostral and caudal) defined the SN region in the sagittal direction (Fig. 1). Automatic and manual shimming procedures were performed. The total acquisition time was approximately 30 minutes.

### Spectra analysis

The 3D MRSI raw data were analyzed using LCModel 6.2-2B (S. W. Provencher), which analyzes an in-vivo spectrum as a linear combination of a set of in-vitro model spectra (basis data set) [3], [4]. To improve the spectral fit quality, the basis data set was optimized by including pathology-specific metabolites [7], [8] and a macromolecule spectrum. The model spectra of 24 metabolites were generated using VeSPA 0.6.0 [9]. The chemical shifts and J coupling constants were taken from the literature [10], [11] and an online database [12]. Metabolite-nulled spectra were acquired from the SN region of 10 volunteers using an additional inversion pulse in the PRESS sequence with TE = 30 ms, TR = 1350 ms, and TI = 410 ms. The resulting averaged macromolecule spectrum was included in the LCModel analysis.

**Figure 2:**
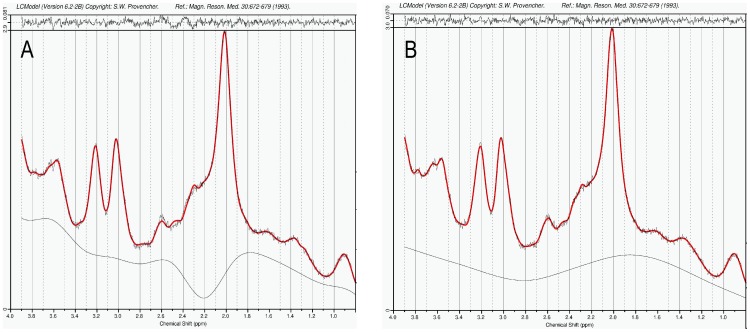
LCModel fitting results of the averaged spectrum from the rostral SN region in the control group with standard basis data set (A) and optimized basis data set (B). Shown are the in-vivo spectra, the fitted spectra with baseline, and the residuals. A) Incomplete spectrum fit (mainly in the region between 2.2 and 2.8 ppm) with clear residuals and humpy baseline, B) improved spectrum fit with closely reproduced in-vivo spectrum and residuals at the noise level.

We validated the optimized basis data set by comparing spectral fitting outputs from the standard basis set with adjusted basis data set. Spectra fitted using standard basis set resulted in clear residuals and humpy baselines. With the use of the optimized basis data set for spectral fitting, the LCModel fits reproduced the in-vivo spectra closely, resulting in residuals at the noise levels that did not show considerable chemical-shift dependence (Fig. 2).

Metabolite concentrations were not corrected for T_1_ and T_2_ relaxation effects, for coil loading or by scaling on water signal.

In addition to individual spectra with low signal/noise ratios (SNR), group-averaged spectra with clearly increased SNR were evaluated for the rostral and caudal voxels from free-induction decay raw data allowing a more accurate assessment, especially in metabolites at lower concentrations.

### Statistics

Statistical analysis was performed using IBM SPSS Statistics 20.0. Due to the low sample size (<50) the Shapiro-Wilk normality test was used to verify the normal distribution of metabolite concentrations in both groups. Afterwards, means and standard deviations (SD) were calculated. Paired t-tests were performed to test for differences between the rostral and caudal metabolite concentrations within the group. In a two-sample t-test, the metabolite concentrations were tested for between-group differences. Post-hoc tests with a Bonferroni-Holm correction for multiple comparisons were applied. The significance level was set to p<0.05.

## Results

### Spectra analysis

The full width at half maximum (FWHM) was estimated to be 0.09±0.02 ppm for the control group and 0.10±0.03 ppm for the patient group. The spectral quality for the PD patients and controls was therefore assessed to be comparable.

Furthermore, there were no differences found in SNR as well as in FWHM between hemispheres. However, significant differences were estimated between the rostral and caudal regions. Therefore, the metabolite concentrations were averaged over both hemispheres but given separately for each SN region.

### Individual spectra

From the individual spectra, only the concentrations of the three main metabolites NAA, Cho, and Cr (Table 1) were estimated with Cramer-Rao lower bounds (CRLB, estimated error of metabolite quantification) less than 20%, which is recommended for reliable results [13]. Low-concentration metabolites such as Glu, Gln, GABA, GSH, Ins, HVA and dopamine showed larger CRLB values mainly caused by insufficient SNR values between 6 and 12, as estimated by LCModel. These metabolites were therefore classified as practically undetectable in individual data [13].

**Table 1:** Individual concentrations [arbitrary units] of the three main metabolites NAA, choline, and creatine in PD patients and age-matched controls.

	Controls (n = 24)			PD patients (n = 21)			p value^2^	
	rostral	caudal	p value^1^	rostral	caudal	p value^1^	rostral	caudal
S/N	9.1±1.5	7.9±1.4	<0.001	8.4±2.0	7.4±1.8	<0.001	0.085	0.178
FWHM (ppm)	0.09±0.02	0.09±0.02	0.638	0.10±0.03	0.09±0.03	0.028	0.033	0.817
NAA+NAAG	57.9±10.8	49.8±9.6	<0.001	47.7±17.5	42.6±13.8	0.005	0.002	0.006
GPC+PCh	5.5±1.2	6.5±1.6	<0.001	4.8±2.0	5.9±2.1	<0.001	0.034	0.114
Cr+PCr	30.3±5.3	29.4±6.3	0.299	28.9±11.4	25.9±9.9	0.016	0.476	0.052

^1^ Significance values from paired t-test between the rostral and caudal values within the group.

^2^ Significance values from two-sample t-test between both groups.

In the control group, the SD for the concentrations of the three main metabolites NAA, Cho, and Cr varied between 17.5 and 24.6%, which are considered to be in the normal range for individual differences. In the PD group, the SDs are larger at between 32.4 and 41.7%.

In both groups, we found significantly higher NAA (controls p<0.001, PD patients p = 0.005) and lower Cho (both p<0.001) concentrations in the rostral region than in the caudal SN regions. Only in PD patients was there a significantly higher rostral than caudal Cr concentration (controls p = 0.299, PD patients p = 0.016).

On the other hand, we found a significant NAA decrease in both SN regions (rostral p = 0.002, caudal p = 0.006) for PD patients as compared with controls. The concentrations of Cho and Cr were also, but not significantly, reduced in PD patients after application of the Bonferroni-Holm correction.

### Averaged spectra

To overcome the inaccuracies in metabolite quantification due to insufficient SNR of individual spectra, group-averaged spectra for the rostral and caudal slices were also analyzed. With the clearly increased SNR values between 39 and 46, a more reliable estimation of lower-concentration metabolites could be achieved with CRLB less than 20%. In accordance with individual findings, clear in-group differences between the rostral and caudal SN regions were found for NAA, Cho, Glu+Gln, Ins, and GABA concentrations for both groups (Table 2). For Cr, GSH and dopamine, these differences were only detected in the PD group.

**Table 2:** Metabolite concentrations [arbitrary units], estimated using the averaged spectra of PD patients and age-matched controls.

	Controls (n = 24)		PD patients (n = 21)	
	rostral	caudal	rostral	caudal
S/N	46	41	41	38
FWHM (ppm)	0.107	0.095	0.119	0.095
NAA+NAAG	50.2 4%	40.7 4%	41.0 4%	31.9 3%
GPC+PCh	5.4 2%	6.3 2%	5.2 3%	5.6 2%
Cr+PCr	28.0 3%	27.2 3%	25.0 4%	21.8 3%
Ins+Gly	27.2 6%	21.8 8%	21.9 8%	19.1 9%
Glu+Gln	10.1 37%	5.0 72%	14.1 27%	13.9 23%
GABA+Hcs	43.6 11%	42.6 11%	49.2 8%	48.3 7%
GABA	21.1 18%	18.4 18%	31.8 15%	21.7 15%
GSH	20.7 6%	20.2 6%	14.0 14%	11.9 11%
Dopamine	12.5 17%	12.1 17%	7.8 37%	2.8 78%
HVA	7.2 23%	9.2 17%	11.8 20%	11.2 17%

Also given are the Cramer-Rao lower bounds [%].

**Figure 3:**
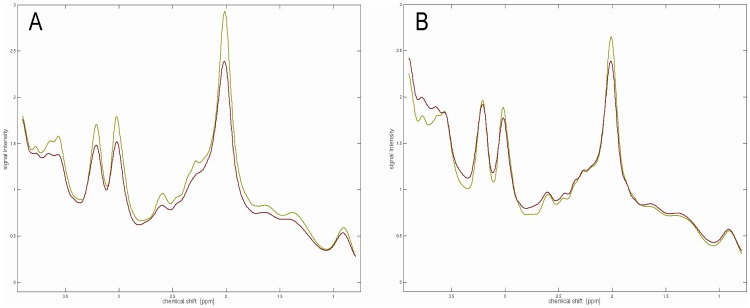
Averaged spectra from the rostral (A) and caudal (B) SN regions of PD patients (red) and controls (green). Beside significant differences in the signal intensities of the three main metabolites NAA, creatine, and choline between both groups, there are clear differences in the range from 2.1 to 2.9

The estimated concentrations for NAA, Cho, Cr, Ins, GSH and dopamine were clearly decreased in PD patients compared to controls, whereas Glu+Gln, GABA and HVA were slightly increased (Table 2). These between-group differences are also clearly visible in the averaged spectra of rostral and caudal voxels (Fig. 3). In addition to considerable concentration differences in the three main metabolites NAA, Cr, and Cho, significant alterations exist in the range from 2.1 to 2.9 ppm between both groups. This range is dominated by the complex multiplet resonance patterns of NAA, GSH, Glu, Gln, and GABA.

## Discussion

In this study, an improved quantitative analysis of in-vivo MR spectra from PD patients and a control group was performed. The results are in contradiction to a previous analysis by Emir et al. [14], but they confirm the post-mortem results in the SN region by Gerlach et al. [2] and Sofic et al. [15] as well as with findings from PD rat model based on proteasome inhibition [20].

It should be noted that the FWHM values were similar in both groups allowing comparison of PD patients and controls. Emir et al. [14] also found no differences in FWHM values determined by LCModel between PD patients and controls in the SN region at 7 T. Differences in SNR are due to reduced main metabolite concentrations in PD patients compared to controls but are not a marker for lower spectral quality. Furthermore, Emir et al. [14] found no significant differences in the metabolic profiles from the SN of PD patients and controls. One reason for this could be the larger voxel size (6×13×13 mm^3^ vs. 6×6×7 mm^3^), which includes both the rostral and the caudal parts of the SN. We found significant differences between the rostral part (dominated by the SN pars reticulata) and the caudal part (dominated by the SN pars compacta) [16] in both groups, as expected from anatomical features. Additionally, these differences are also in good agreement with post-mortem results by Gerlach et al. [2].

Moreover, the gradients of metabolite concentrations between the rostral and caudal regions have the same direction in PD patients and controls in both individual data and averaged spectra. This applies to all metabolites, regardless of whether they are increased or decreased for pathological reasons.

For clinical application, the acquisition time of the used 3D MRSI method had to be acceptable for PD patients. Therefore, a repetition time TR of 1350 ms was used, which caused a T_1_ weighting. Additionally, an echo time TE of 30 ms led to a slight T_2_ weighting, but the metabolite levels were not corrected for relaxation effects. Interestingly, we found very similar concentrations to Kirov et al. [17]. They estimated the absolute concentrations of NAA, Cr, Cho, and Ins, which were approximately the same as our values (arbitrary units) if a constant factor of 5 was applied.

This study gives the necessary biochemical explanation for our phenomenological findings published recently [5], [6]. However, the advantage of the phenomenological investigation using the Syngo software in previous studies [5], [6] is that the spectra evaluation can performed directly on the MR scanner, which is important for clinical application because it is the only approved method. It is reproducible, user-independent, very fast, and robust [18]. Moreover, it could be shown that significant differentiation between patients with idiopathic PD and atypical Parkinsonism was possible in individual subjects [5]. However, problems in quantification are caused by complex multiplet resonance patterns, overlapping metabolites, variations in line shape and underlying baseline variations, which cannot be fitted accurately using only a single peak, as was done with the Syngo software package. Therefore, the estimated rostral-to-caudal NAA/Cr ratios in previous studies [5], [6] could only be evaluated as phenomenological values and did not allow pathophysiological interpretation. Furthermore, in previous studies, the standardized automatic baseline correction was performed using a 6^th^-order polynomial in the calculation range between 0 to 4.3 ppm (excluded ranges: 3.5–4.15 ppm, 2.9–3.4 ppm, 1.85–2.72 ppm, 1.2–1.6 ppm, 0.7–1.1 ppm). However, metabolites such as NAA, GSH, Glu, Gln, and GABA show complex multiplet resonance patterns, especially in the range from 2.1 to 2.9 ppm with low signal amplitudes caused by J couplings and low metabolite concentrations. The automatic baseline correction draws these multiplets partially to the zero line. On the other hand, this 6^th^ order polynomial raises the Cr signal and possibly also the Cho signal, resulting in overestimation due to integration of the signal at 3.0 ppm. With the new findings presented in this work, we can provide a pathophysiological explanation for this. However, the presented method is not applicable for routine clinical application due to the unacceptable time and effort required.

Using the optimized basis data set for the LCModel analysis yielded robust and reliable values for the metabolite concentrations in both groups. The improved fitting quality was reflected by the closely reproduced the in-vivo spectra, flatness of the resulting baseline and negligible residuals at the noise level without a considerable chemical-shift dependence.

The main limitation of our study is that the clinical diagnosis could not be confirmed by post-mortem histological examinations. Additionally, the very small voxel size necessitated by the anatomy of the SN resulted in low SNR of individual spectra. The relatively high iron content of SN caused broader intrinsic line widths. Both facts lead to high standard deviations and obscured differences in low concentration metabolites at the individual level. However, by calculating averaged spectra we overcome the problems of insufficient SNR and could confirm post-mortem results [2], [15] by comparing PD patients with controls. Only aspartate was practically undetectable.

Motor symptoms of PD result from progressive degeneration of dopamine-producing neurons within the SN pars compacta [1]. Mitochondrial dysfunction and oxidative stress are a possible cause of PD pathogenesis. Therefore, reduced NAA (putative marker of viable neurons), Cr (marker for impaired energy metabolism due to mitochondrial dysfunction), GSH (marker for oxidative stress) [19] and dopamine concentrations are expected in PD patients. Moreover, elevated GABA/Glu concentrations are expected for innervation of GABA/Glu-ergic neurons. In this study we could non-invasively detect these expected de- and increases in vivo for the first time using an optimized 3D MRSI method. Furthermore, the observed decrease in NAA, Cho, Cr, Ins, GSH and dopamine and slightly increase in Glu+Gln and GABA are in good agreement with post-mortem results from Gerlach et al. [2] and Sofic et al. [15] as well as with findings from PD rat model based on proteasome inhibition [20]. Only in the case of HVA did we find a slight elevation that is in contradiction to the post-mortem data [2]. This finding might be explained by the fact that dopamine, given as a drug in PD, finally degrades to HVA by the action of the enzymes monoamine oxidase and catechol-O-methyl transferase.

To the best of our knowledge, these are currently the only direct metabolic findings for the SN region identified through non-invasive imaging. The signal overlaps of NAA, GSH, Glu, Gln, and GABA, which occur in short-echo-time data acquisitions, were overcome by increasing the SNR by averaging spectra and spectral fitting using an improved basis data set that includes pathology-specific metabolites.

In conclusion, this study provides a method by which it should be possible to monitor disease progression and to validate pathophysiological explanation for metabolic changes in PD, which could help to better understand the disease and to develop new therapy options with neuron protection and long-term delay of disease progression.
